# Influence of dietary carbon on mercury bioaccumulation in streams of the Adirondack Mountains of New York and the Coastal Plain of South Carolina, USA

**DOI:** 10.1007/s10646-012-1003-3

**Published:** 2012-10-26

**Authors:** Karen Riva-Murray, Paul M. Bradley, Lia C. Chasar, Daniel T. Button, Mark E. Brigham, Barbara C. Scudder Eikenberry, Celeste A. Journey, Michelle A. Lutz

**Affiliations:** 1 U.S. Geological Survey, 425 Jordan Road, Troy, NY 12180 USA; 2 U.S. Geological Survey, Columbia, SC USA; 3 U.S. Geological Survey, Tallahassee, FL USA; 4 U.S. Geological Survey, Columbus, OH USA; 5 U.S. Geological Survey, Mounds View, MN USA; 6 U.S. Geological Survey, Middleton, WI USA

**Keywords:** Methylmercury bioaccumulation, Carbon source, Stable isotopes, Macroinvertebrates, Fish, Trophic ecology, Streams and rivers, Lower food web

## Abstract

We studied lower food webs in streams of two mercury-sensitive regions to determine whether variations in consumer foraging strategy and resultant dietary carbon signatures accounted for observed within-site and among-site variations in consumer mercury concentration. We collected macroinvertebrates (primary consumers and predators) and selected forage fishes from three sites in the Adirondack Mountains of New York, and three sites in the Coastal Plain of South Carolina, for analysis of mercury (Hg) and stable isotopes of carbon (δ^13^C) and nitrogen (δ^15^N). Among primary consumers, scrapers and filterers had higher MeHg and more depleted δ^13^C than shredders from the same site. Variation in δ^13^C accounted for up to 34 % of within-site variation in MeHg among primary consumers, beyond that explained by δ^15^N, an indicator of trophic position. Consumer δ^13^C accounted for 10 % of the variation in Hg among predatory macroinvertebrates and forage fishes across these six sites, after accounting for environmental aqueous methylmercury (MeHg, 5 % of variation) and base-N adjusted consumer trophic position (Δδ^15^N, 22 % of variation). The δ^13^C spatial pattern within consumer taxa groups corresponded to differences in benthic habitat shading among sites. Consumers from relatively more-shaded sites had more enriched δ^13^C that was more similar to typical detrital δ^13^C, while those from the relatively more-open sites had more depleted δ^13^C. Although we could not clearly attribute these differences strictly to differences in assimilation of carbon from terrestrial or in-channel sources, greater potential for benthic primary production at more open sites might play a role. We found significant variation among consumers within and among sites in carbon source; this may be related to within-site differences in diet and foraging habitat, and to among-site differences in environmental conditions that influence primary production. These observations suggest that different foraging strategies and habitats influence MeHg bioaccumulation in streams, even at relatively small spatial scales. Such influence must be considered when selecting lower trophic level consumers as sentinels of MeHg bioaccumulation for comparison within and among sites.

**Electronic supplementary material:**

The online version of this article (doi:10.1007/s10646-012-1003-3) contains supplementary material, which is available to authorized users.

## Introduction

Mercury (Hg) in freshwater biota often is strongly linked to dissolved methylmercury (MeHg) concentrations (Mason et al. 2000; Hammerschmidt and Fitzgerald 2006; Chasar et al. 2009; Ward et al. 2010), and organism trophic position (Kidd et al. 1995; Mason et al. 2000; Ward et al. 2010). The type of carbon at the base of the food web is a potentially important control that is less well described. In lakes, greater Hg bioaccumulation has been reported in autochthonous (algal-carbon based) pelagic food webs than in more allochthonous (terrestrial carbon-based) littoral food webs (Kidd et al. 1995; Montgomery et al. 2000; Power et al. 2002; Stewart et al. 2008, Chetelat et al. 2011). This may be due to active methylation in the fine particulate matter (Montgomery et al. 2000), efficient uptake of aqueous MeHg by phytoplankton (Stewart et al. 2008; Chetelat et al. 2011), and (or) the greater assimilation, by consumers, of autochthonous than allochthonous carbon due to the greater nutritional value of the former (Stewart et al. 2008).

Differences in basal-carbon source also may influence Hg bioaccumulation in lotic food webs, but this possibility is less well-documented or understood (Ward et al. 2010; Jardine et al. 2012). Macroinvertebrate and fish consumers in forested streams rely to varying degrees on carbon originating in the stream channel and carbon entering from terrestrial habitats (Cummins and Klug 1979). The relative importance of these autochthonous and allochthonous food sources varies as a function of the consumer’s feeding strategy and habitat, the stream reach’s geographic location and position in the drainage network, and the amount of shading and organic matter inputs from the riparian zone (Cummins and Klug 1979; Vannote et al. 1980; Minshall et al. 1985; Lau et al. 2009).

Consumer ^13^C:^12^C ratios can reveal differences in the primary carbon source at the base of aquatic food webs (Peterson and Fry 1987; Jardine et al. 2006), because the carbon stable isotope is conserved with little (e.g., 0.3 to 1.0  ‰) trophic fractionation (McCutchan et al. 2003). The δ^13^C of terrestrial detritus (primarily from C_3_ plants) in temperate streams worldwide is about −28.2 ± 0.2 ‰ (SE; Finlay 2001), while the δ^13^C of autochthonous organic matter varies widely (LaZerte and Szalados 1982; Rounick et al. 1982; France 1995; Finlay 2004). Differences in δ^13^C between phytoplankton and terrestrially-derived detritus have been used to distinguish autochthonous from allochthonous carbon sources in lakes, and to relate them to consumer Hg concentrations (Kidd et al. 1995; Montgomery et al. 2000; Power et al. 2002; Stewart et al. 2008; Chetelat et al. 2011). However, this application is more challenging in small to mid-sized streams, where the algal base portion of the food web is typically associated with periphyton. Periphyton includes various amounts of dead algae, bacteria, fungi, and animal material, in addition to living algae (Vander Zanden et al. 1997), often resulting in δ^13^C that is indistinguishable from that of terrestrial detritus (France 1995). Periphyton δ^13^C also exhibits large spatial and temporal variation (Rosenfeld and Roff 1992; France 1995; Finlay 2004; Hill and Middleton 2006). For example, France (1995) report attached algal δ^13^C ranging from −40 to −20 ‰ across 803 published studies, and Finlay (2004) report δ^13^C ranging from −44 ‰ to −23 ‰ within a single stream network. Despite these limitations, comparisons of biofilm MeHg concentrations to those in detritus collected from the same site have revealed much higher concentrations in biofilm (Tsui et al. 2009), indicating a greater potential for MeHg bioaccumulation by consumers of periphytic algae than by consumers of detritus. Recently, Jardine et al. (2012) used a gradient approach (following Rasmussen (2010)) across 60 streams in New Brunswick, Canada, to examine relations between periphyton δ^13^C and both δ^13^C and Hg in consumers. In acidic streams, they found higher levels of Hg in consumers that were trophically linked to periphyton than in consumers associated with terrestrial carbon. However, no difference in Hg bioaccumulation between these consumer groups was apparent in neutral waters. Additional studies are needed to clarify the potential influence of dietary carbon source on Hg bioaccumulation in streams over an extended range of geographic and ecologic settings, and encompassing a variety of geochemical and food web characteristics.

In this study, we assess the potential for dietary carbon source to influence MeHg bioaccumulation in relatively small to mid-sized streams (in catchments ranging from about 18 to 80 km^2^ in area). These streams are located in the Fishing Brook basin and the McTier Creek basin. The Fishing Brook basin is a portion of the Upper Hudson River basin, and is located in the central Adirondack Mountain region of New York. The McTier Creek basin is a portion of the Edisto River basin, and is located in the Sand Hills portion of South Carolina’s inner Coastal Plain. Both areas are sensitive to the atmospheric deposition of Hg from distant sources (Driscoll et al. 2007; Evers et al. 2007; Glover et al. 2010; NYDOH 2010; SCDHEC 2010). Previously, Riva-Murray et al. (2011) showed increasing biotic MeHg with consumer trophic position in both areas, and a strong positive relation with aqueous (filtered) MeHg (FMeHg) concentration across the topographically-heterogeneous Fishing Brook study area. Here, we (1) compare carbon isotope signatures and MeHg concentrations of sympatric primary consumers having distinct feeding strategies, (2) quantify the contribution of dietary carbon to variation in primary consumer MeHg concentration at the stream reach scale, after accounting for trophic position, (3) describe variation among sites in carbon signatures and MeHg concentrations of secondary consumers, and (4) quantify the contribution of dietary carbon to observed spatial variation in secondary consumer MeHg concentration, after accounting for consumer trophic position and stream water FMeHg.

## Methods

### Site selection

**Fig. 1:**
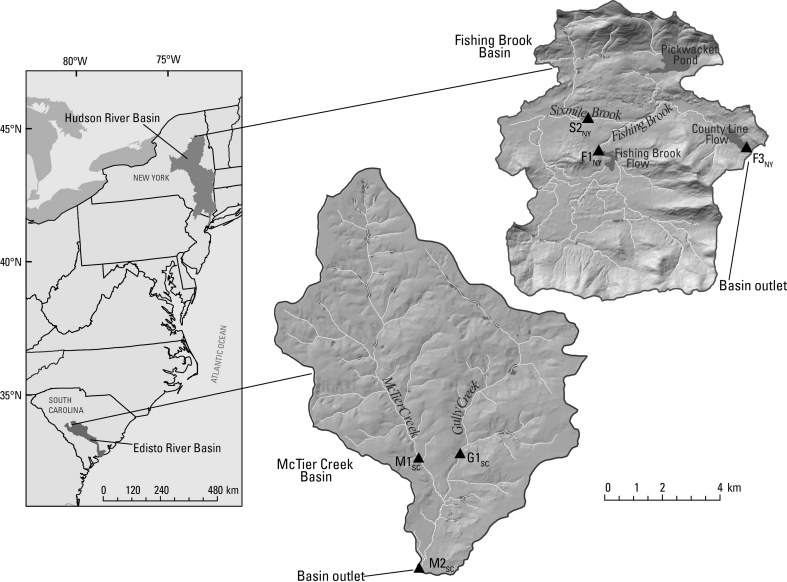
Map of study area showing site locations (*dark triangles*). Site names are provided in Table 1

Three sites each were selected in the Fishing Brook and McTier Creek basins (Fig. 1, Table 1). Sites were selected to capture the range of landscape variability within these mid-sized (<80 km^2^) basins, to include the outlet of each basin (sites F3_NY_ and M2_SC_, respectively), and to ensure sufficient numbers of primary and secondary consumers. Characteristics of study sites varied within and among the two basins with respect to channel width, channel depth, and degree of channel shading by riparian tree canopy (Table 1, Online Resource #1), and consequently, with respect to the potential for benthic primary production. Two of the six sites had full sun exposure, two were partially shaded, and two were heavily shaded. Sixmile Brook (S2_NY_) is fully exposed due to the dominance of herbaceous vegetation and deciduous shrubs in a wide riparian zone. Gully Creek (G1_SC_) is a wide, shallow reach with little riparian shading. In contrast, the two other SC sites, McTier Creek at Monetta (M1_SC_) and McTier Creek at New Holland (M2_SC_) have narrow stream channels covered by over-arching tree canopy. Fishing Brook near Long Lake (F1_NY_), and Fishing Brook (County Line Flow) near Newcomb (F3_NY_) are both partly shaded. Although F3_NY_ has a very wide channel, the photic zone is largely limited to a 1–3 m wide littoral zone shaded by mixed evergreen- deciduous canopy that overhangs the channel’s edge.

**Table 1:** List of sites from which macroinvertebrates and (or) fish were collected in NY and SC, basin and reach characteristics

Site abbreviation, Site name, and Station identifier	Basin characteristics		Wetted channel morphology Mean Range (n)		Dominant riparian vegetation, and relative amount of canopy shading of benthic habitat
	Basin area (km^2^)	Wetland amount (%)	Width (m)	Depth (m)	
F1_NY_ Fishing Brook @ 28 N near Long Lake, NY 0131199010	27.1	5.8	2.9 2.2–4.7 (10)	0.23 0.04–0.60 (44)	Shrub-scrub, evergreen and deciduous trees; partial canopy (moderate shade)
S2_NY_ Sixmile Brook near Long Lake, NY 0131199022	17.7	13.0	19.8 5.6–25.7 (6)	0.23 0.01–1.28 (90)	Shrub-scrub and herbaceous, open canopy (no shade)
F3_NY_ Fishing Brook (County Line Flow) near Newcomb, NY 0131199050	65.6	8.2	284.7 177.6–406.3 (8)	1.75 0.23–2.90 (56)	Evergreen and deciduous trees; partial canopy (moderate shade)
G1_SC_ Gully Creek on Shoals Road near Monetta, SC 3345100813509	25.9	5.3	58.6 25.0–61.0 (4)	0.47 0.01–3.00 (45)	Herbaceous, deciduous and evergreen trees; mostly open canopy (little shade)
M1_SC_ McTier Creek (Road 209) near Monetta, SC 02172300	40.5	5.1	3.8 2.6–6.1 20	0.14 0.01–0.50 185	Deciduous and evergreen trees; closed canopy (full shade)
M2_SC_ McTier Creek at New Holland, SC 02172305	79.4	6.4	5.4 3.4–6.4 20	0.19 0.01–0.61 200	Deciduous and evergreen trees; closed canopy (full shade)

### Stream water chemistry sampling and processing

Water samples were collected from six to 25 times under base flow conditions across the growing seasons during 2007–2009. The growing season is defined here as May 1 through October 30 in NY, and as April 1 through November 30 in SC. Each sample was collected from the approximate centroid of flow. Ultra-trace-level clean techniques, as described in Brigham et al. (2009), were used to collect and process samples for FMeHg analysis. The reporting limit for FMeHg was 0.04 ng/L. Field measurements of pH were obtained with a portable probe, and water samples were collected and analyzed for dissolved organic carbon (DOC) by persulfate oxidation and for total unfiltered nitrogen (N_tot_) by alkaline persulfate digestion.

### Consumer organism sampling

Macroinvertebrates and fishes were collected four to eight times (median 6 times) during 2007–2009, mainly during spring (defined here as May and June in NY, and April and May in SC) and summer (defined here as July–Sept in NY and June–Aug in SC), with a few samples collected during fall months (i.e., October in NY and November in SC). Macroinvertebrates were collected from various locations throughout each sampling reach (but primarily from edge habitat in nonwadable sections) by hand picking and netting. Three samples of each taxon, with each composite sample comprised of at least 15 specimens, were collected whenever possible. Within taxa, specimens covering large ranges in sizes, approximating different life stages, were placed in separate composite samples. Three caddisfly taxa (order Trichoptera) and one mayfly taxon (order Ephemeroptera) that are broadly considered ‘primary consumers’, but that have different feeding habits and diets, were targeted for collection. All were common and abundant in the NY sites, and two were also present in the SC sites (no primary consumers were common or abundant in SC). Two of the caddisfly taxa were northern casemaker caddisflies (family: Limnephilidae, tribes: Stenophylacini and Limnephilini) that are mainly shredding detritivores with some differences in habitat and feeding modes. Stenophylacini (mainly *Hydatophylax* spp.) make cases primarily of twigs and bark, inhabit areas of slow currents on stream margins, and consume mainly dead vascular plant tissue (wood, bark, Wiggins 1996). Limnephilini (mainly *Limnephilus* spp.) make various types of cases; those we collected were primarily hut-shaped cases of evergreen needles and pieces of macrophyte leaves; the Limnephilini can be more herbivorous than the Stenophylacini (Wiggins 1996). We refer to Stenophylacini as ‘stick-builder’ caddisflies, and to Limnephilini as’hut-builder’ caddisflies. The third targeted caddisfly taxon was Hydropsychidae, which are lotic, collector-filterers that build silk nets to capture particulate organic matter (Wiggins 1996). The Hydropsychidae consume particulate organic matter that can include diatoms, bacteria and protozoans in the seston, and animal material (Carlough and Meyer 1989; Benke and Wallace 1997). Heptageniidae (flathead mayflies), the fourth targeted primary consumer taxon, graze on periphyton scraped from woody and rocky substrates (Merritt and Cummins 1996). All four of these taxa are important prey for secondary and higher-order consumers. *Tipula* sp. (Trichoptera: Tipulidae), a shredding detritivore (Merritt and Cummins 1996), also was collected from SC sites to augment low numbers of other primary consumers found. Dragonflies (order Odonata) in families Aeshnidae (darners) and Libellulidae (common skimmers) were targeted secondary consumer (predatory) macroinvertebrates. Aquatic larvae of both families are engulfing predators, with different primary habitats and feeding modes. Darners climb on woody debris and macrophytes and are active stalkers of prey. Common skimmers are ‘lie in wait’ predators that sprawl on the stream bottom in depositional areas and among debris and macrophytes and (Merritt and Cummins 1996; Needham et al. 2000). Both are known to consume the targeted primary consumers and are, themselves, prey of fish collected for this study. Fish were collected from throughout wadable reaches, and along edge habitat of nonwadable reaches, by electrofishing and netting, as described in Riva-Murray et al. (2011). Selected minnows (family Cyprinidae) were targeted for regional comparisons because of their similar habitats and feeding strategies. These were common shiners (*Luxilus cornutus*) in NY and mainly yellowfin shiner (*Notropus lutipinnis*) in SC. Both are omnivorous generalist feeders (Smith 1985; Rohde et al. 1994). Whole fish were rinsed in deionized water and frozen as individual specimens or (more often) as composites of 2–14 (median 7) similarly-sized fish, as described in Riva-Murray et al. (2011).

### Biota Hg and stable isotope analyses

We analyzed macroinvertebrates for MeHg because MeHg-to-THg ratios vary widely among macroinvertebrate taxa (Mason et al. 2000). Whole-body fish samples were analyzed for THg because MeHg comprises greater than 95 % of Hg in fish that consume some animal material (Huckabee et al. 1979; Grieb et al. 1990; Bloom 1992). Henceforth, Hg in biota refers to MeHg, either directly measured (macroinvertebrates), or measured as THg and assumed to be primarily MeHg (fish). Prior to analysis, biological samples were freeze-dried to constant weight and ground to a fine powder with a stainless steel ball mill (Retsch MM200). Macroinvertebrate samples were analyzed for MeHg at the U.S. Geological Survey (USGS) Wisconsin Mercury Research Laboratory by cold-vapor atomic fluorescence spectroscopy after extraction by dilute nitric acid per (Hammerschmidt and Fitzgerald 2005). Laboratory precision for triplicates was 7.5 % (±7.1 SE), and percent recoveries for MeHg concentration in blind submissions of standard reference material were 90.9 % (±5.60 % SE), 83.5 % (±2.79 % SE), and 93.0 % (±3.80 % SE), for NIST 2976; NRCC DOLT-3 and TORT-2, respectively. Fish samples were analyzed for THg at the Trace Element Research Laboratory (Texas A&M University, College Station, Texas) using USEPA Method 7473 (combustion and atomic absorption using a Milestone DMA-80 direct Hg analyzer). The mean percent recoveries for THg concentration in blind submissions of standard reference material were 90.5 % (±5.90 % SE), 98.2 % (±3.77 % SE), and 118.5 % (±10.30 % SE) for NIST 2976; NRCC DOLT-3 and TORT-2, respectively. Additional quality assurance details are available in Beaulieu et al. (2012).

Samples were analyzed for δ^15^N and δ^13^C at the Stable Isotope Geochemistry Laboratory of Florida State University’s National High Magnetic Field Laboratory (Tallahassee, Florida). Samples were analyzed with a ThermoQuest NC2500 Elemental Analyzer interfaced with a Finnegan MAT Delta Plus XP isotope ratio mass spectrometer. Isotope ratios were measured relative to reference gases and calibrated to known carbon and nitrogen standards [δ^13^C_PDB_ and δ^15^N_air_, ranging from −12.7 to −32.1 ‰ and −5.3 to 2.5 ‰ respectively]. Additional QA/QC included blind duplicates and blind standard reference material samples (glutamic acid, USGS-40, NIST-8573) included in sample runs (approximately two for every ten samples). Precision and accuracy for isotopic ratios were <0.4 ‰, and generally <0.2 ‰ for carbon and nitrogen, respectively. Additional quality assurance details are available in Beaulieu et al. (2012).

### Data analysis

Growing-season means for FMeHg, pH, DOC, and Total N were calculated as the grand means of seasonal means, and the growing season mean for FMeHg was used in regression analysis. Statistical comparisons among sites were performed on summer-collected samples, which were the most numerous across all sites. Data were pooled across sites within each basin for regional comparisons. Chemical comparisons were done by analysis of variance followed by Tukey’s HSD test on appropriately-transformed data (base-10 logarithm for FMeHg and DOC, and square root for Total N), These analyses were conducted in SAS software, version 9.2 (SAS Institute, Cary, NC). FMeHG values below the reporting limit (0.04 ng/L) were treated as half the detection limit for statistical analysis and plotting.

Consumer samples were pooled across seasons and years after preliminary tests indicated no significant or consistent temporal variation in δ^13^C within taxa nested within sites. Differences in δ^13^C among primary consumer taxa were tested for statistical significance by permutational analysis of variance (PERMANOVA), with unrestricted permutation of raw data (Anderson 2001), using Primer-E + PERMANOVA software, version 1.0.3 (Clarke and Gorley 2006) This approach provides exact tests and does not require an assumption of normally distributed errors (Anderson and Ter Braak 2003). Monte Carlo simulation (Anderson 2001) was used to generate p-values for pair-wise tests having fewer than 9000 possible permutations.

Nonparametric distance-based linear regression (DistLM, Legendre and Anderson 1999, McArdle and Anderson 2001) in Primer E + PERMANOVA software (Clarke and Gorley 2006) was used to develop models of consumer Hg. Primary consumer Hg concentration was modeled with δ^15^N and δ^13^C within each of the three NY sites, from which all four primary consumer taxa were collected. Multi-site models of secondary consumer Hg concentration were developed with aqueous MeHg (i.e., FMeHg), consumer Δδ^15^N (i.e., base consumer-adjusted δ^15^N), and δ^13^C. Three multi-site models were produced – one for all six sites, one for only NY sites, and one for only SC sites. DistLM (Legendre and Anderson 1999; McArdle and Anderson 2001) was used to perform 9,999 permutations of residuals from a Euclidian-distance matrix. Predictor variables were input sequentially into the model to determine whether δ^13^C explained a significant proportion of Hg variation among samples only after considering the other explanatory variables. Data for mean growing season FMeHg were log-transformed. Directions of influence of explanatory variables in each model were obtained through classical multiple regression. Regression analyses were done with SigmaPlot software, version 12 (Systat Software, Inc.).

## Results and discussion

### Stream water characteristics

The Fishing Brook and McTier Creek basins exhibited broad regional differences in stream water chemistry, as well as differences in the degree of spatial heterogeneity in stream water chemistry within each basin (Online Resource #2). Stream water of the Fishing Brook basin was near neutral, with growing season mean pH ranging from 6.2 at S2_NY_ to 6.7 at F3_NY_. In contrast, McTier Creek stream water was significantly more acidic (*F* = 57.19, *p* < 0.0001), with pH ranging from 4.7 at G1_SC_ to 5.4 at M2_SC_. The two basins also differed significantly in mean growing season concentrations of FMeHg (*F* = 16.97, *p* = 0.0003) and DOC (*F* = 22.95, *p* < 0.0001), but they had similar N_tot_ concentrations (*p* = 0.14). FMeHg concentrations ranged from 0.16 ng/L at F3_NY_ to 0.54 ng/L at S2_NY_ in the Fishing Brook basin, and from 0.11 ng/L at M2_SC_ to 0.13 ng/L at G1_SC_ in the McTier Creek basin. DOC concentrations ranged from 8.4 mg/L at F3_NY_ to 13.3 mg/L at S2_NY_ in the Fishing Brook basin and from 5.1 mg/L at G1_SC_ to 7.4 mg/L at M2_SC_ in the McTier Creek basin. Over all six sites, total N ranged from 0.31 mg/L at G1_SC_ to 0.53 mg/L at M1_SC_. Stream water chemistry varied spatially across the Fishing Brook basin, where S2_NY_ had significantly higher concentrations of FMeHg (*F* = 19.17, *p* = 0.0004), DOC (*F* = 11.25, *p* = 0.0018), and N_tot_ (*F* = 10.04, *p* = 0.0041) than F1 and F3. In contrast, the only difference among sites within the McTier Creek basin was slightly higher FMeHg at G1_SC_ than at M2_SC_ (*F* = 5.19, *p* = 0.021). The greater spatial heterogeneity of bioavailable FMeHg in Fishing Brook compared to McTier Creek is consonant with the more heterogeneous landscape of the Upper Hudson Basin (Bradley et al. 2011; Riva-Murray et al. 2011; Burns et al. 2012). The differences within the Fishing Brook basin and between the two study areas, particularly in the amount of MeHg that is potentially available for biological uptake, support the inclusion of FMeHg in our consumer Hg models.

### Mercury and stable isotopes in sympatric primary consumers

**Fig. 2:**
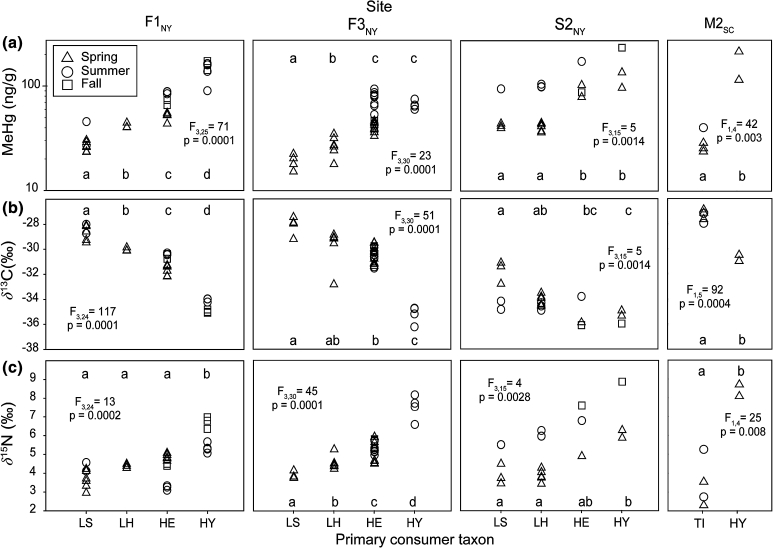
**a** Methylmercury concentration, **b** carbon isotope ratio (δ^13^C), and **c** nitrogen isotope ratio (δ^15^N) in primary consumers collected from three NY sites and one SC site during spring, summer, and fall. Macroinvertebrate samples are composites collected from throughout each reach. Taxa names of invertebrate larvae are as follows: HE, flat-head mayflies (Heptageniidae); HY, net-spinner caddisflies (Hydropsychidae); LS, stick-builder northern case-maker caddisflies (Limnephilidae); LH, hut-builder northern case-maker caddisflies (Limnephilidae); TI, craneflies (Tipulidae: *Tipula* spp.). Taxa with *same letter above symbols* within sites are not significantly different (*p* > 0.05). *F* statistic (*subscripts* are degrees of freedom) and *p*-values are based on analyses using all taxa within each site

The number of primary consumer composite samples collected per feeding group ranged from two to 20 among all three Fishing Brook sites and M2_SC_; few samples of any primary consumers were collected from G1_SC_ and M1_SC_. Detailed sample data can be found in Beaulieu et al. (2012). Primary consumers differed with respect to MeHg concentrations, δ^13^C, and δ^15^N within each of the four sites from which multiple primary consumers were collected (Fig. 2), but patterns were generally consistent among sites. At all four sites, shredders had the lowest concentrations, and filterers, or filterers and scrapers, had the highest concentrations. Differences also were apparent within the shredder feeding group at two of the three NY sites, where MeHg concentrations were higher in hut-builder caddisflies than in stick-builder caddisflies. Within-site δ^13^C patterns among primary consumers were generally the inverse of the MeHg pattern. Shredders had the most enriched δ^13^C (which was the most similar to typical detrital δ^13^C), and filterers, or filterers and scrapers, had the most depleted δ^13^C (which was the most distinct from typical detrital δ^13^C). Scraper δ^13^C was depleted relative to one of the shredders (i.e., stick-builder caddisflies) at all three NY sites, and also was depleted relative to hut-builder caddisflies at F1_NY_.

The relative depletion or enrichment of these sympatric primary consumers is consistent with published feeding and dietary characteristics (Merritt and Cummins 1996; Wiggins 1996) and with findings of more enriched δ^13^C in shredders (i.e., more similar to δ^13^C of terrestrial detritus) than in scrapers from relatively small (<100 km^2^), less productive streams (e.g., Rounick et al. 1982; Finlay 2001; Jardine et al. 2012). For example, Jardine et al. (2012) reported more enriched δ^13^C in shredder stoneflies (Pteronarcydae) than in scraper mayflies (Heptageniidae) and beetles (Psphenidae) in New Brunswick (Canada) streams. Jardine et al. (2012) also found a strong positive relation between scraper δ^13^C and periphyton δ^13^C across their study streams. The δ^13^C differences among consumers in our study are likely due in large part to differences in the dietary importance of terrestrial versus algal carbon among these taxa. However, other factors cannot be ruled out. For example, the more depleted δ^13^C of the flathead mayflies and hydropsychid caddisflies may be at least partly due to feeding on periphyton in more rapidly flowing areas where CO_2_ supply to benthic algae is enhanced (Finlay et al. 1999). At our sites, filterers were limited to riffles, the shredders were limited to quiescent depositional areas along channel margins, and the scrapers were found in various habitats, in flowing waters and quiescent sections. Additional variation may be attributed to the assimilation of seston carbon by filterers, as was reported by Tsui and Finlay (2011), who found δ^13^C of Hydropsychidae to be similar to that of seston in Minnesota (U.S.) streams. Filterer δ^13^C may also reflect the assimilation of carbon from upstream habitats, in contrast to a more localized δ^13^C signature of scrapers and other taxa (Finlay et al. 2002). Thus, δ^13^C differences among these consumers may reflect localized (within-reach) differences in food source that are associated with foraging habitat, as well as differences in consumption and assimilation of carbon from in-stream or terrestrial sources.

Some of the observed variation among taxa in MeHg concentration may be due to trophic position differences, even though these organisms are all broadly considered ‘primary consumers’. The δ^15^N pattern within these four sites (Fig. 2c) indicates variation in trophic position among taxa sampled for this study. The more enriched δ^15^N of the filterers, and in scrapers at some sites may be partly due to functional omnivory in the collected macroinvertebrate taxa (i.e., Hydropsychidae and Heptageniidae, respectively; Merritt and Cummins 1996; Wiggins 1996, Benke and Wallace 1997; Furderer et al. 2003). The higher δ^15^N of the net-spinning caddisflies also could be related to greater consumption of bacteria and protozoans from the seston (Carlough and Meyer 1989; Benke and Wallace 1997; Finlay et al. 2002). The latter possibility is especially likely at F1_NY_ and F3_NY_, where the Hydropsychidae were limited to flowing waters downstream of open water bodies.

At sites with a wide range of dietary carbon source characteristics (based on consumer δ^13^C), we found that dietary assimilation of more depleted δ^13^C is associated with higher consumer MeHg (Table 2). As much as 34 % of the variation in primary consumer MeHg within these sites is explained by δ^13^C, after accounting for trophic position (as indicated by δ^15^N). The exception was S2_NY_, where we found little variation in δ^13^C (all relatively depleted) and MeHg (all relatively high). Jardine et al. (2012) report higher Hg in macroinvertebrates associated with algal consumption (i.e. more autochthonous route of exposure) than those linked to terrestrial food sources (i.e., more allochthonous route of exposure) in low-pH streams, but they did not observe these differences in circumneutral streams. Our results indicate that enhanced Hg bioaccumulation also may be associated with more depleted δ^13^C (associated with greater herbivory in at least some cases) in near-neutral streams (F1_NY_, S2_NY_, and F3_NY_), as well as in acidic streams (M2_SC_). Explanations may include higher MeHg in periphyton than in detritus, as was found by Tsui et al. (2009), and that may result from several factors, including active uptake of aqueous MeHg by algae (Moye et al. 2002) and methylation by periphytic bacteria (Guimaraes et al. 2006; Tsui et al. 2010).

**Table 2:** Distance-based linear models of methylmercury in primary consumers for three NY sites

Site (*n*)	Variable	AIC	*p*	*R* ^2^ (cumulative)
F1_NY_ (28)	δ^15^N (+)	−12.4	0.0001	0.42
	δ^13^C (−)	−34.9	0.0001	0.76
F3_NY_ (34)	δ^15^N (+)	−12.5	0.0004	0.37
	δ^13^C (−)	−13.5	0.10	0.42
S2_NY_ (18)	δ^15^N (+)	−26.5	0.0001	0.81
	δ^13^C (−)	−24.5	0.99	0.81

Variables were entered sequentially into each model, in order of appearance in the table. *AIC* Akaike’s information criterion. Direction of influence for each variable is indicated as positive (+) or negative (−)

A potential link between MeHg bioaccumulation and dietary carbon source characteristics (indicated by δ^13^C) in low-trophic level consumers has implications for use of these organisms in mercury monitoring. There is much interest in the use of lower trophic level taxa as mercury ‘sentinels’, because of numerous advantages over higher-order consumers such as predatory fish (Jardine et al. 2005). We found significant variation in Hg concentrations among lower trophic level consumers within the same stream reach, and a potential link to diet and habitat after controlling for the effects of trophic position. Based on these results, careful selection of sentinel taxa is warranted, and lumping across feeding groups is contraindicated. In our sampling, we composited samples from throughout the stream reach. A stronger linkage with δ^13^C may have been apparent with separation of samples according to within-reach location and habitat. Future studies that consider these stream reach habitat differences may provide additional insight into the controls on MeHg bioaccumulation in streams.

### Site-to-site patterns of mercury and stable isotopes in secondary consumers

**Fig. 3:**
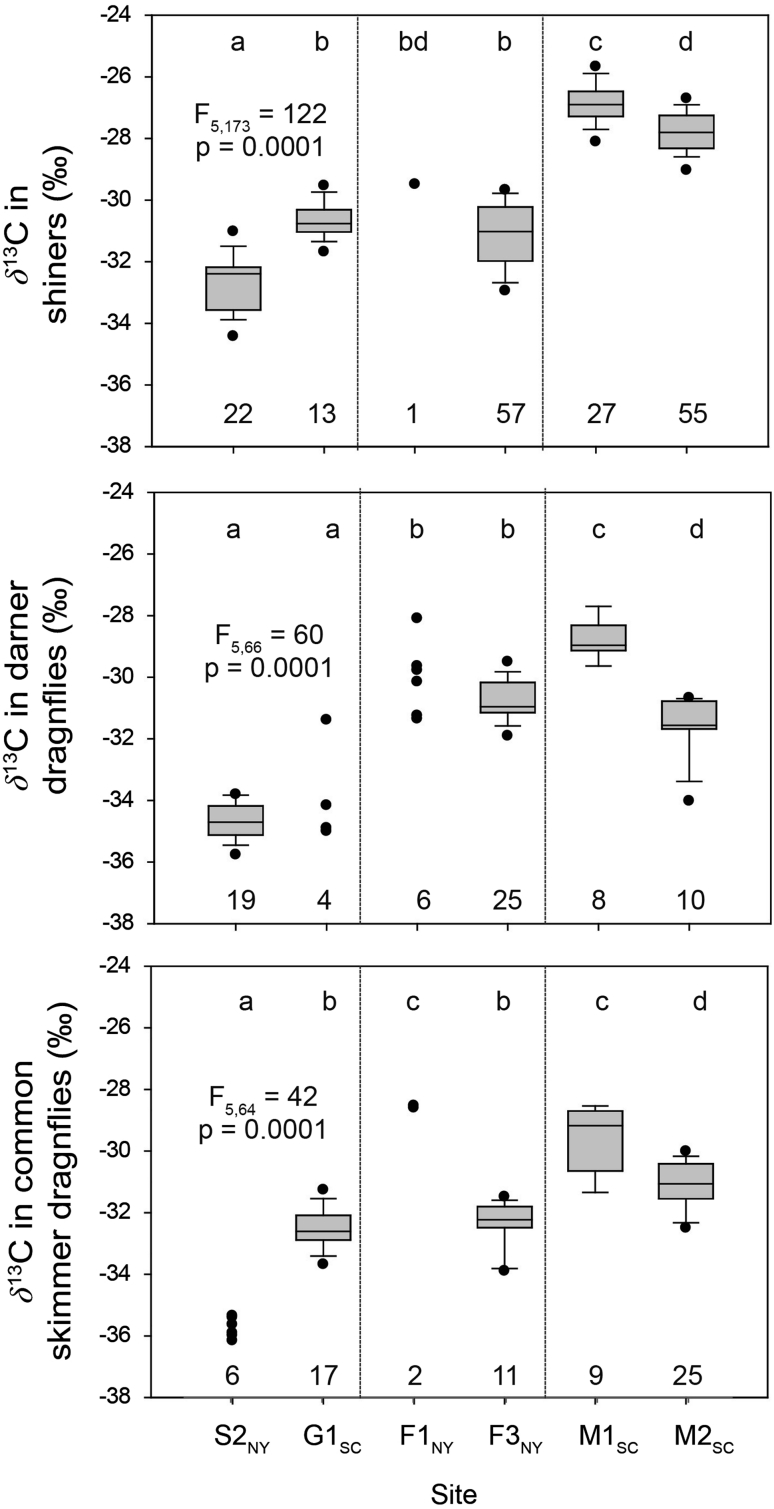
Carbon stable isotope ratios (δ^13^C) in shiners (Cyprinidae: *Luxilus cornutus* and *Notropis* spp.), darner dragonflies (Odonata: Aeshnidae), and common skimmer dragonflies (Odonata: Libellulidae) collected from sites in New York and South Carolina. Site names are provided in Table 1; locations are shown on Fig. 1. Numbers of samples are indicated above x-axes. Groups with same letter above box are not significantly different (*p* > 0.05). *Vertical dotted lines* separate open canopy sites (*left*) from partially shaded sites (*middle*) and heavily shaded sites (*right*). *Boxes* represent interquartile range with median line; *lower* and *upper whiskers* represent 10th and 90th percentiles, respectively; and *dots* (associated with *boxes*) indicate 5th and 95th percentiles. *F* statistic (*subscripts* are degrees of freedom) and *p*-values are based on analyses using all groups

The number of samples collected per secondary consumer taxon per site from one to 57; the median number of samples was 14 (Online Resource #3). Hg and Δδ^15^N statistics for these sites are provided in Online Resource #3, and detailed data are provided in Beaulieu et al. (2012). Mean Hg in the selected secondary consumers ranged from 155 ng/g in common skimmer dragonflies collected from G1_SC_ to 693 ng/g in shiners collected from S2_NY_. Significant variation in Hg and trophic position among sites within these and other secondary consumers has been reported previously (Riva-Murray et al. 2011). In the current study, we also found significant site-to-site variation in δ^13^C (Fig. 3) of selected secondary consumers, with significantly more depleted δ^13^C at S2_NY_ than the other sites, and with M1_SC_ and M2_SC_ having enriched δ^13^C in some taxa. This pattern may be due at least partly to site-to-site differences in the potential for benthic primary production, and the importance of autochthonous versus allochthonous carbon. Enhanced benthic primary production is likely in the exposed reaches of S2_NY_ and G1_SC_ (i.e., sites with the most depleted δ^13^C), compared with the partially shaded reaches of F1_NY_ and F3_NY_ (i.e. sites with generally intermediate δ^13^C) and the heavily shaded reaches of M1_SC_ and M2_SC_ (i.e., sites with the most enriched δ^13^C). Rounick et al. (1982) also reported more depleted consumer δ^13^C in open-canopy stream reaches compared with shaded reaches, and attributed this pattern to greater autochthony in the former. However, as indicated for sympatric primary consumers in our study, δ^13^C variation in dietary carbon source characteristics among sites also may be influenced by other factors not considered here, such as site-to-site differences in gradient and habitat that may affect water velocity.

Dietary carbon source, indicated by consumer δ^13^C, explained a significant portion of the variation in consumer Hg for shiners, darner dragonflies and common skimmer dragonflies, after accounting for site-to-site variation in both FMeHg and base-adjusted trophic position (Table 3). Together, FMeHg and Δδ^15^N explained 19 % of the variation in consumer Hg across all six sites. The addition of δ^13^C as an explanatory variable in the model increased the amount of variation explained to 30 %. The negative influence of δ^13^C in each model indicates a link between more depleted δ^13^C (i.e., more distinct from typical δ^13^C in detritus of terrestrial origin) and greater accumulation of Hg by consumers. This result indicates that factors controlling the form of dietary carbon can potentially be important additional controls on secondary consumer Hg bioaccumulation across these two geographically-distinct study areas. Similarly, δ^13^C accounted for significant additional variation in Hg of secondary consumers within the FB_NY_ and MC_SC_ basins (Table 3), suggesting that factors that influence δ^13^C, such as site-to-site differences in benthic primary production, also may be important controls on Hg bioaccumulation at these smaller spatial scales. Higher primary production in periphyton may also contribute to higher consumer Hg due to active uptake of MeHg from water (Moye et al. 2002), as well as by providing suitable environments for microbial growth and Hg methylation and accumulation (Guimnaraes et al. 1998; Cleckner et al. 1999; Desrosiers et al. 2006; Guimaraes et al. 2006; Tsui et al. 2010; deWit et al. 2012). Rapid algal growth has been related to lower MeHg concentrations (i.e., growth dilution) in some settings (Pickardt et al. 2002; Hill and Larsen 2005). However, the low nutrient levels in the oligotrophic forested streams in our study (Online Resource #2) do not favor rapid algal growth. Somatic growth dilution has also been reported in consumers, whereby the faster growth of those feeding on more nutritive algal carbon results in lower tissue Hg concentrations (Karimi et al. 2007). The importance of somatic growth dilution in consumers at Fishing Brook or McTier Creek basin sites cannot be evaluated with the data collected for the current study.

**Table 3:** Distance-based linear models of mercury in secondary consumers from the Fishing Brook basin (NY) and the McTier Creek basin (SC)

Variable	AIC	*p*	*R* ^2^ (cumulative)
All sites (number of samples = 301)			
FMeHg (+)	−11.5	0.0002	0.05
Δδ^15^N (+)	−87.0	0.0001	0.26
δ^13^C (−)	−128.3	0.0001	0.36
Fishing Brook basin (number of samples = 135)			
FMeHg (+)	11.2	0.0001	0.19
Δδ^15^N (+)	−20.0	0.0001	0.36
δ^13^C (−)	−29.4	0.001	0.42
McTier Creek basin (number of samples = 166)			
FMeHg (+)	−75.5	0.0019	0.06
Δδ^15^N (+)	−91.1	0.0001	0.15
δ^13^C (−)	−113.2	0.0001	0.27

Riva-Murray et al. (2011) reported a link between landscape characteristics and Hg bioaccumulation in the Fishing Brook basin, but observed no Hg-landscape relation in the McTier Creek basin. Based on the findings of Jardine et al. (2012), a relation between conditions favorable to algal production and MeHg bioaccumulation might be expected in the McTier Creek basin due to this basin’s greater acidity (Online Resource #2) and possible enhanced MeHg transfer across biological membranes (Mason et al. 1996). Thus, the amount of canopy cover could help explain some of the spatial variation in Hg bioaccumulation across settings, such as the Coastal Plain, that have less variability in fluvial MeHg concentration or landscape characteristics. It is also possible that a greater response to sunlight exposure might be seen in the more acidic streams of the western Adirondacks (Baldigo et al. 2009) than in the more neutral streams of the Fishing Brook basin.

## Summary and conclusions

Our study, conducted in small to mid-sized streams of New York’s Adirondack Mountains and South Carolina’s Coastal Plain, provides evidence that dietary carbon signatures of primary and secondary consumers vary within and among these streams and that diets dominated by more depleted carbon are associated with greater Hg bioaccumulation at multiple spatial scales. Four major findings were noted. First, we describe significant variation in δ^13^C among sympatric primary consumers and a pattern of more depleted δ^13^C associated with more herbivorous feeding strategies. Second, we demonstrate that this small-scale variation in δ^13^C can account for significant additional variation in MeHg concentration among sympatric primary consumers, beyond the influence of trophic position alone. Third, we describe significant site-to-site variation in secondary consumer δ^13^C, and a pattern of more depleted δ^13^C associated with more open-canopy sites that have greater potential for primary production. Forth, we demonstrate that δ^13^C also accounts for significant broader-scale spatial variation in Hg concentration among secondary consumers, after accounting for differences in potentially-bioavailable aqueous MeHg and differences in trophic position. Overall, we found potentially greater Hg bioaccumulation associated with more herbivorous diets and associated with sites having greater potential for primary production. The influence of MeHg supply and trophic position on Hg bioaccumulation is well supported in the literature. Our study contributes to a growing body of evidence that the source of dietary carbon is an important additional control on Hg bioaccumulation in streams. Thus, factors that influence primary production of forested streams, like canopy cover, could account for some of the spatial variability in Hg bioaccumulation at multiple scales. In addition, the foraging strategies and habitats of lower food web consumers are important to consider when selecting sentinel taxa for Hg monitoring of streams.

## Electronic supplementary material

Below is the link to the electronic supplementary material.

Supplementary material 1 (PDF 305 kb)
