# Supplementary material for: Influence of dietary carbon on mercury bioaccumulation in streams of the Adirondack Mountains of New York and the Coastal Plain of South Carolina, USA
Source: Ecotoxicology. 2012 Oct 26;22(1):60–71. doi: 10.1007/s10646-012-1003-3 (PMC3526734; doi:10.1007/s10646-012-1003-3)
Supplement: Supplementary file 1 — Supplementary material 1 (PDF 305 kb) [file 10646_2012_1003_MOESM1_ESM.pdf]

Riva-Murray_Ecotoxicology_Online_Resource_1.pdf

Influence of dietary carbon on mercury bioaccumulation in streams of the Adirondack Mountains of New York and the Coastal Plain of South Carolina, USA

Karen Riva-Murray*, Paul M. Bradley, Lia C. Chasar, Daniel T. Button, Mark E. Brigham, Barbara C. Scudder Eikenberry, Celeste A. Journey, and Michele A. Lutz
*U.S. Geological Survey, 425 Jordan Road, Troy, N.Y., USA, 12180
krmurray@usgs.gov

Plate S1. Photographs of stream reaches showing differing amounts of canopy cover and riparian shading of the benthic habitat from which macroinvertebrates and fish were collected. (a) Heavily shaded sites, with dense tree canopy over narrow channels; mean width is 3.8 m at $M1_{SC}$ and 5.4 m at $M2_{SC}$; photo of $M1_{SC}$ was taken during non-growing season; (b) partially shaded sites $F1_{NY}$ and $F3_{NY}$; mean channel width of $F1_{NY}$ is 2.9 m; although much of the actual channel of $F3_{NY}$ is very wide (mean channel width 285 m) and exposed, the photic benthic habitat is limited to 1-3 meters from the channel edge, which is shaded by dense tree cover along the banks; (c) more fully exposed sites $S1_{NY}$ and $G1_{SC}$; mean channel widths are 19.8 m at $S1_{NY}$ and 58.6 m at $G1_{SC}$; the photograph of $G1_{SC}$ shows dead trees within the stream channel.

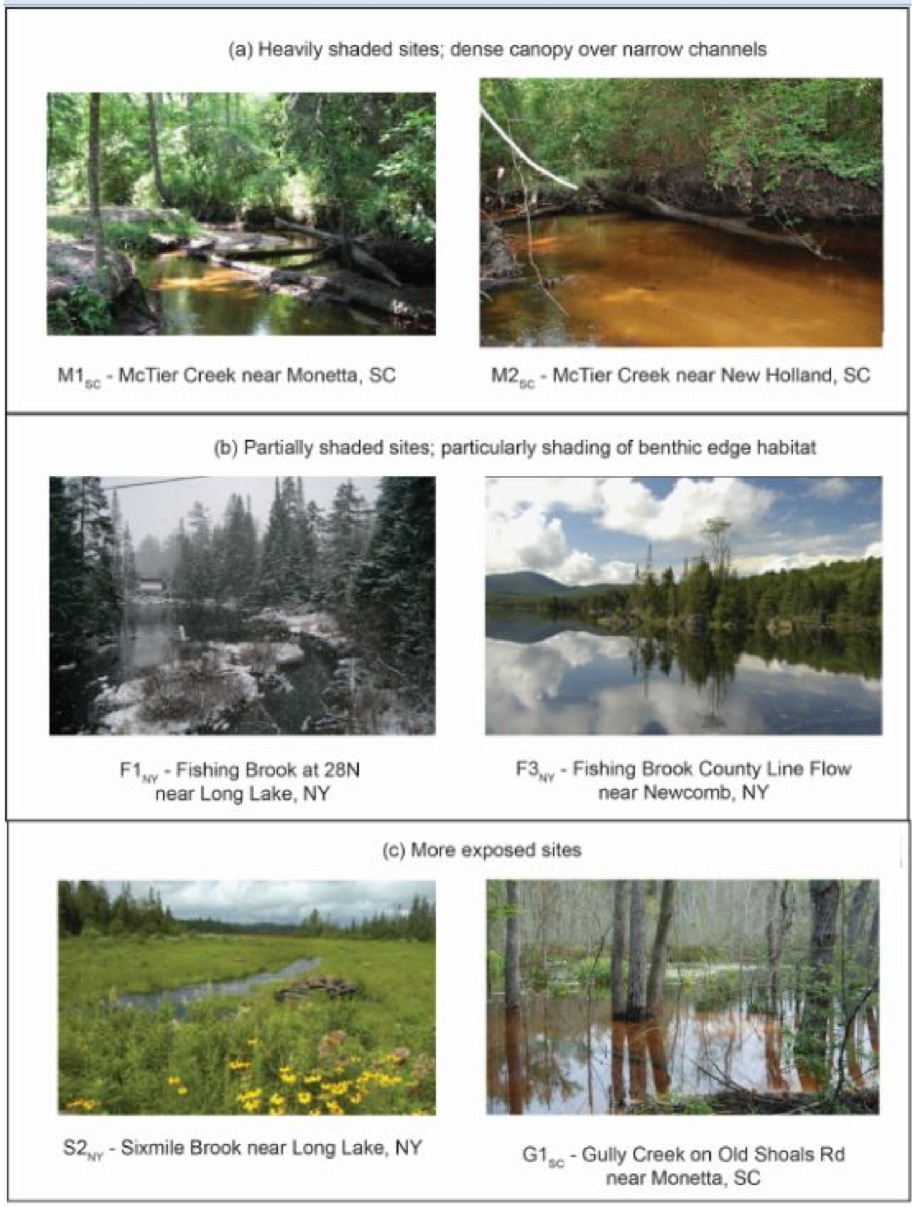

Suppl Mtrls (not cover letter - place cover letter in "comments")

Riva-Murray_Ecotoxicology_Online_Resource_2.pdf
Influence of dietary carbon on mercury bioaccumulation in streams of the Adirondack Mountains of New York and the Coastal Plain of South Carolina, USA

Karen Riva-Murray*, Paul M. Bradley, Lia C. Chasar, Daniel T. Button, Mark E. Brigham, Barbara C. Scudder Eikenberry, Celeste A. Journey, and Michele A. Lutz
*U.S. Geological Survey, 425 Jordan Road, Troy, N.Y., USA, 12180
krmurray@usgs.gov

Figure S1. Selected chemical characteristics of study sites. Samples were collected under base flow conditions across the growing seasons of 2007-2009. Site names are provided in table 1.

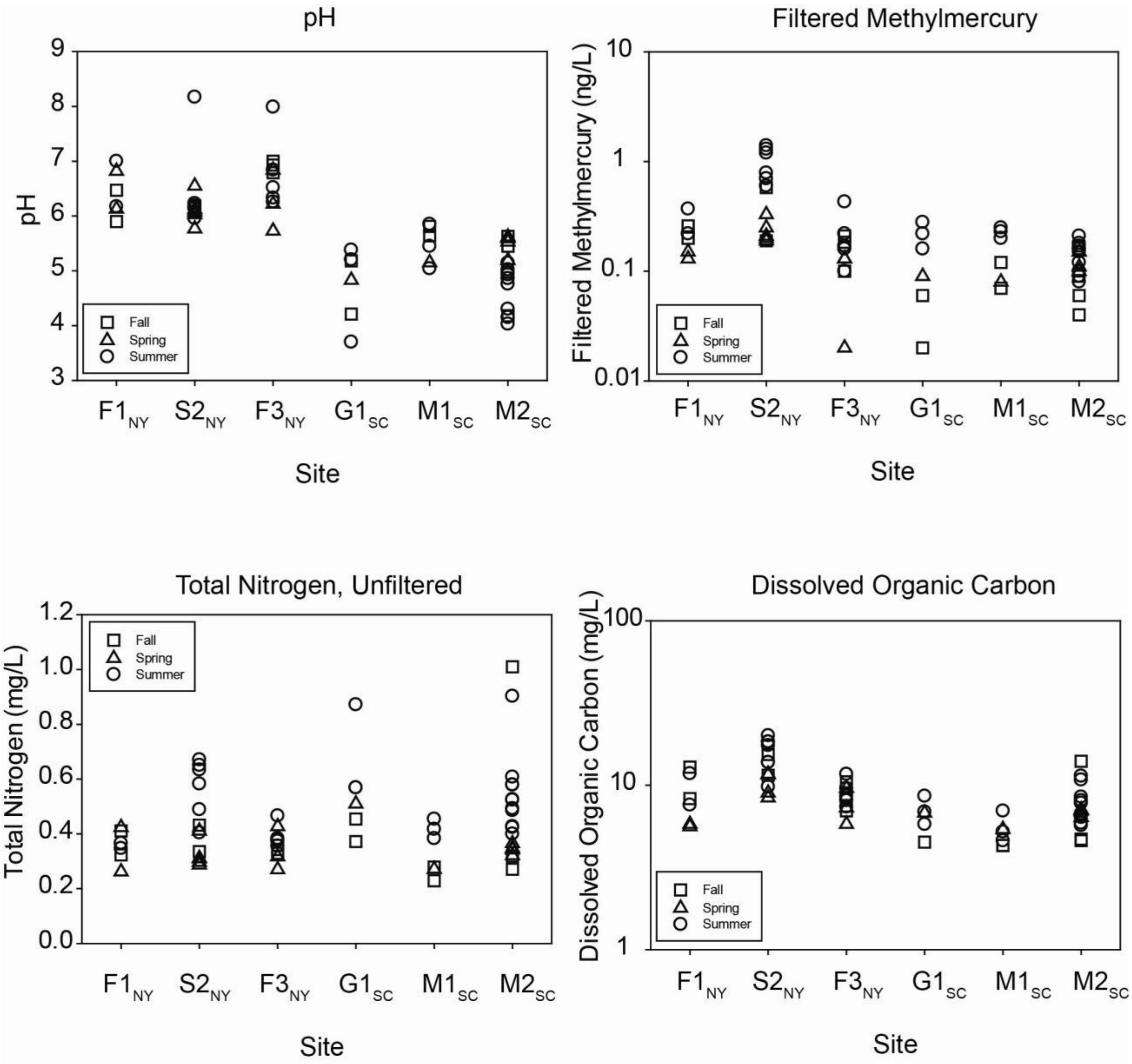

Riva-Murray_Ecotoxicology_Online_Resource_3.pdf
Influence of dietary carbon on mercury bioaccumulation in streams of the Adirondack Mountains of New York and the Coastal Plain of South Carolina, USA
Karen Riva-Murray*, Paul M. Bradley, Lia C. Chasar, Daniel T. Button, Mark E. Brigham, Barbara C. Scudder Eikenberry, Celeste A. Journey, and Michele A. Lutz
*U.S. Geological Survey, 425 Jordan Road, Troy, N.Y., USA, 12180
krmurray@usgs.gov

Table S1. Mercury (Hg) concentrations (total Hg, THg for fish and methylmercury, MeHg for invertebrates) and base-N adjusted nitrogen stable isotope ratios ($\Delta\delta^{15}N$) of selected secondary consumers collected from six sites. Shiner species collected from NY sites were common shiner (*Luxilus cornutus*); those collected from SC sites were mainly yellowfin shiner (*Notropis lutipinnis*). Two families of dragonfly larvae (Odonata) were collected: Aeshnidae (darner dragonflies) and Libellulidae (common skimmer dragonflies) family Libellulidae). Values are means, with standard deviation in parentheses; n, number of samples collected (most samples are composites of multiple specimens).

Site	Shiners			Darner dragonflies			Common skimmer dragonflies		
	n	THg (ng/g dw)	$\Delta\delta^{15}N$	n	MeHg (ng/g dw)	$\Delta\delta^{15}N$	n	MeHg (ng/g dw)	$\Delta\delta^{15}N$
$F1_{NY}$	1	252.0	3.52	6	177.3 (14.2)	2.67 (0.23)	2	174.5 (9.19)	2.62 (0.13)
$S2_{NY}$	22	693 .0 (127.3)	5.25 (0.59)	18	305.3 (49.1)	4.33 (1.29)	6	272.2 (79.8)	2.0 (0.49)
$F3_{NY}$	57	428.1 (133.4)	5.77 (0.53)	25	192.9 (29.3)	3.34 (0.55)	11	154.6 (25.1)	2.57 (0.55)
$G1_{SC}$	16	562.9 (154.8)	8.92 (0.28)	4	318.3 (27.9)	5.42 (0.35)	17	210.1 (76.7)	5.53 (0.52)
$M1_{SC}$	27	511.9 (119.2)	5.91 (0.62)	8	423.1 (124.9)	6.32 (0.52)	9	274.6 (93.9)	6.24 (0.41)
$M2_{SC}$	55	490.3 (122.3)	6.34 (0.81)	9	541.0 (171.6)	4.93 (0.80)	25	281.7 (82.6)	5.30 (0.70)
